# Health-related quality of life in primary cutaneous B-cell lymphoma following local radiotherapy

**DOI:** 10.1016/j.jdin.2024.07.002

**Published:** 2024-07-30

**Authors:** Alice Heger, Khaled Elsayad, Christian Kandler, Jan Siats, Michael Oertel, Christopher Kittel, Hans Theodor Eich

**Affiliations:** Department of Radiation Oncology, University Hospital of Muenster, Muenster, Germany

**Keywords:** diffuse large B-cell lymphoma-leg type, follicle center lymphoma, primary cutaneous marginal zone lymphomas, quality of life, radiotherapy

*To the Editor:* In this prospective cohort, the influence of local radiotherapy (RT) on the quality of life in patients with primary cutaneous B-cell lymphoma (CBCL) is determined and compared before and after radiotherapy.^1^, ^2^, ^3^ Forty-seven patients with primary cutaneous B-cell lymphomas, thereof 18 primary cutaneous marginal zone lymphomas, 22 primary cutaneous follicle center lymphoma, and 7 primary cutaneous diffuse large B-cell lymphoma-leg type (Table I) were treated from 2017 to 2024. The median radiotherapy (RT) dose was 36 Gy (range, 4-50). The median follow-up period was 42 months. The 3-year local control rate was 97 ± 3%, and the median freedom from progression was 93 ± 5% (Fig 1). At the time of analysis, 6 patients had deceased. The treated cases completed the Skindex-29 and European Organization for the Research and Treatment of Cancer Quality of Life C30 (EORTC-QLQ-C30) questionnaire before (first day of radiation) and after treatment (3 months after radiation).^4^^,^^5^

**Table I:** Patient, disease, and treatment characteristics

Characteristic	N (%)
Median age, y (range)	58 (21-87)
≤58 y	24 (51)
>58 y	23 (49)
Sex	
Male	27 (57)
Female	20 (43)
Type of primary cutaneous B-cell lymphoma	
Indolent follicle center	22 (47)
Indolent MZL	18 (38)
DLBCL-leg type	7 (15)
Anatomic site	
Head and neck	19 (40)
Others	28 (60)
T-classification at time RT	
T1	13 (28)
T2	16 (34)
T3	12 (25)
NA	6 (13)
CLIPI scores	
0-1	18 (38)
2-3	19 (40)
Radiotherapy timing	
Upfront RT	35 (75)
Salvage RT	12 (25)
Previous therapies	
Surgery	14 (30)
Doxycycline	12 (25)
Rituximab	7 (15)
Chemotherapy	4 (9)
None	18 (38)
Response	
Yes	45 (96)
CR	31 (66)
PR	14 (30)
No	2 (4)
SD	1 (2)
PD	1 (2)

*CR*, Complete remission; *CLIPI*, cutaneous lymphoma international prognostic index; *DLBCL*, diffuse large B-cell lymphoma; *MZL*, marginal zone lymphoma; *N*, number of radiation courses; *NA*, not available; *PD*, progressive disease; *PR*, partial remission; *RT*, radiotherapy; *SD*, stable disease.

**Fig 1:**
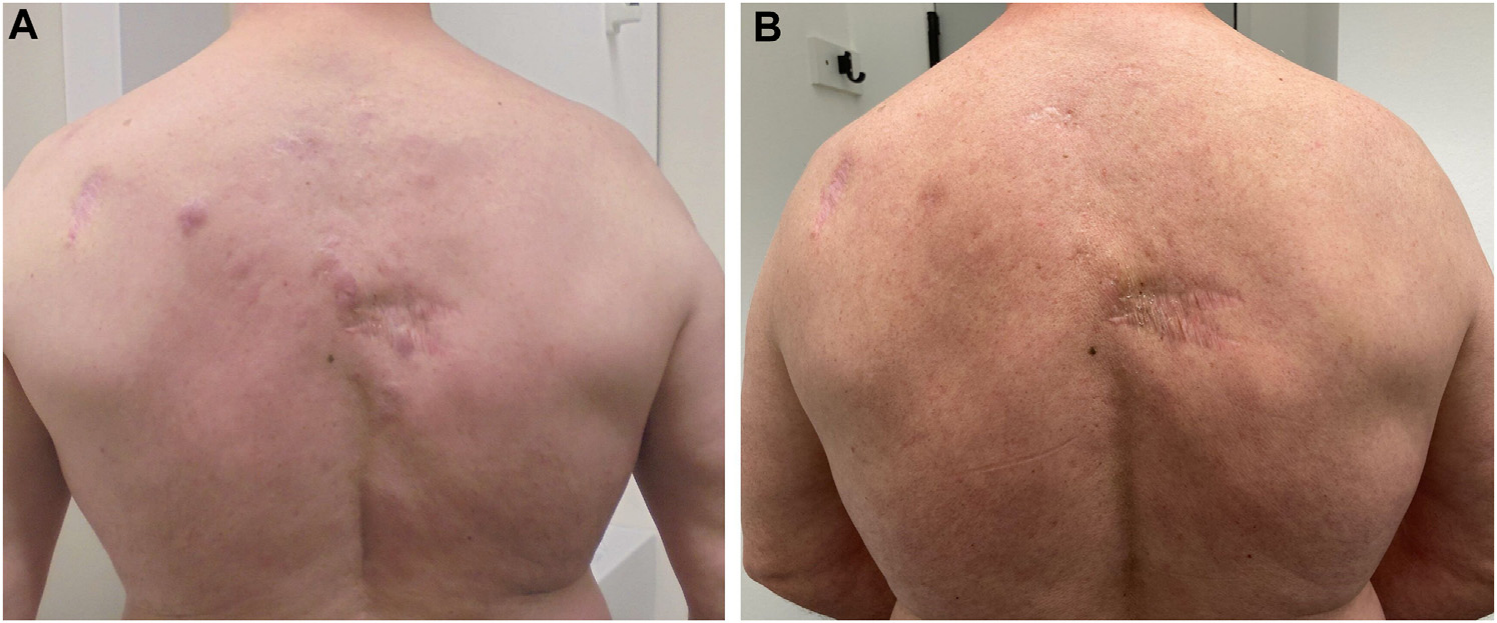
Primary cutaneous marginal zone lymphoma relapse, after surgical resection and anti-CD20 antibody rituximab, presenting with multiple nodules in the trunk. Lesions, before treatment (**A**), and 4 weeks after local radiation therapy with 4 Gy in 2 fractions depicting complete remission of the lesions (**B**).

When comparing Skindex-29 scores before and after RT, Skindex-29 global scores reduced after treatment with a mean difference (mDiff) of 1.33 (*P* = .2). However, we could not detect a significant improvement in subdomains for the Skindex-29 subdomains analysis (*P* > .05). The mDiff in symptoms, emotions, functioning, and fear of side effects was mDiff = 0.56, 0.73, 0, and 0.92, respectively. In subgroup analysis, we could not glimpse any significant discrepancy regarding the type of lymphoma and CLIPI scores. Regarding T-classification, only patients with T3 lesions significantly improved the emotions subscale after treatment (mDiff = −2; *P* = .04). However, the overall Skindex-29 scores significantly improved in younger patients (mDiff = 2.10; *P* = .036). Regarding the site of RT, there was a significant enhancement in Skindex-29 overall scores in locations other than head and neck (mDiff = 2.38; *P* = .017). In subscale analysis, emotional scores (mDiff = 2.29; *P* = .022) were also improved after treatment of lesions outside the head and neck. Radiation dose and response did not influence the Skindex-29 outcomes (*P* > .05). Relapse-free survivors had a trend toward improvement of the Skindex-29 global scores (mDiff = 1.53; *P* = .1) and emotional subscale (mDiff = 1.65; *P* = .1).

We could not detect any significant dissimilarity in the EORTC-QLQ-C30 questionnaire (*P* > .05), such as global health status (mDiff = 0.45) and functional subscales (physical, mDiff = 1.713; role, mDiff = 0.419; social, mDiff = 0.542; and emotional, mDiff = 0.383). In subgroup analysis, we observed no significant difference regarding type of lymphoma, age, and CLIPI scores. Regarding the site and extent of cutaneous manifestations, the physical subscale seems to improve after treatment of head and neck lesions (mDiff = 1.782; *P* = .07) and T3 lesions (mDiff = 1.732; *P* = .08). Patients who received more than 30 Gy have a nonsignificant trend toward emotional distress (mDiff = 1.539; *P* = .1) and a worse global health score subscale (mDiff = 1.543; *P* = .1). On the contrary, there was a trend toward physical worsening in patients who received less than 30 Gy (mDiff = 1.820; *P* = .07). Patients with relapse seem to suffer from worsening social subscales (mDiff = 1.473; *P* = .1).

The present analysis shows that local RT was not associated with the quality-of-life deterioration in CBCL. In contrast, selected patients with CBCL receiving local RT might experience clinically meaningful health-related quality of life changes.

## Conflicts of interest

None disclosed.
